# Expanding the clinical spectrum associated with the *PACS1* p.Arg203Trp mutational hot‐spot: Two additional Italian patients

**DOI:** 10.1002/ajmg.a.62984

**Published:** 2022-10-09

**Authors:** Lucia Pia Bruno, Gabriella Doddato, Margherita Baldassarri, Caterina Lo Rizzo, Sara Resciniti, Mirella Bruttini, Lista Mirjam, Kristina Zguro, Simone Furini, Maria Antonietta Mencarelli, Alessandra Renieri, Francesca Ariani

**Affiliations:** ^1^ Medical Genetics University of Siena Siena Italy; ^2^ Med Biotech Hub and Competence Center, Department of Medical Biotechnologies University of Siena Siena Italy; ^3^ Genetica Medica Azienda Ospedaliera Universitaria Senese Siena Italy

*To the Editor*:

The Schuurs–Hoeijmakers Syndrome (SHMS), also known as *PACS1* neurodevelopmental disorder (PACS1‐NDD), is a rare condition characterized by impaired intellectual development, distinct craniofacial features, and variable additional congenital abnormalities (Tenorio‐Castaño et al., 2021). It is caused by heterozygous pathogenic variants in *PACS1* gene (OMIM# 615009) located on chromosome 11q13 (Simmen et al., 2005). Ethnic background is known to affect the clinical manifestations of the syndrome; this can be due both to the impact of the genetic context and to the exposure to different environmental factors (Dutta, 2019). SHMS worldwide prevalence is unknown and only 61 patients have been published so far (Tenorio‐Castaño et al., 2021). To date, only two missense variants in *PACS1* gene, which affect the same amino acid, have been reported as the cause of SHMS: c.607C>T (p.Arg203Trp) and c.608G>A (p.Arg203Gln) (Figure 1). The first variant is present in all reported cases except for one patient in which it was identified the second variant (Miyake et al., 2018). *PACS1* encodes a trans‐Golgi‐membrane protein, regulating the protein cargo (Scott et al., 2003). This gene is expressed at high levels in the brain during the embryonic period whereas it is down regulated in the postnatal period (Liu et al., 2021). Experiments in zebrafish have documented a role for the PACS1 protein in cranial neural crest migration, explaining the specific facial features in patients (Schuurs‐Hoeijmakers et al., 2012). The mutation is located in the furin cargo binding domain, directly adjacent to a CK2‐binding motif that is essential for *PACS1* autoregulation as well as for interaction with cargo proteins, or for adaptor complexes binding (Scott et al., 2003). Currently, therapies exploiting antisense RNA are under development (Simmen et al., 2005).

**FIGURE 1:**
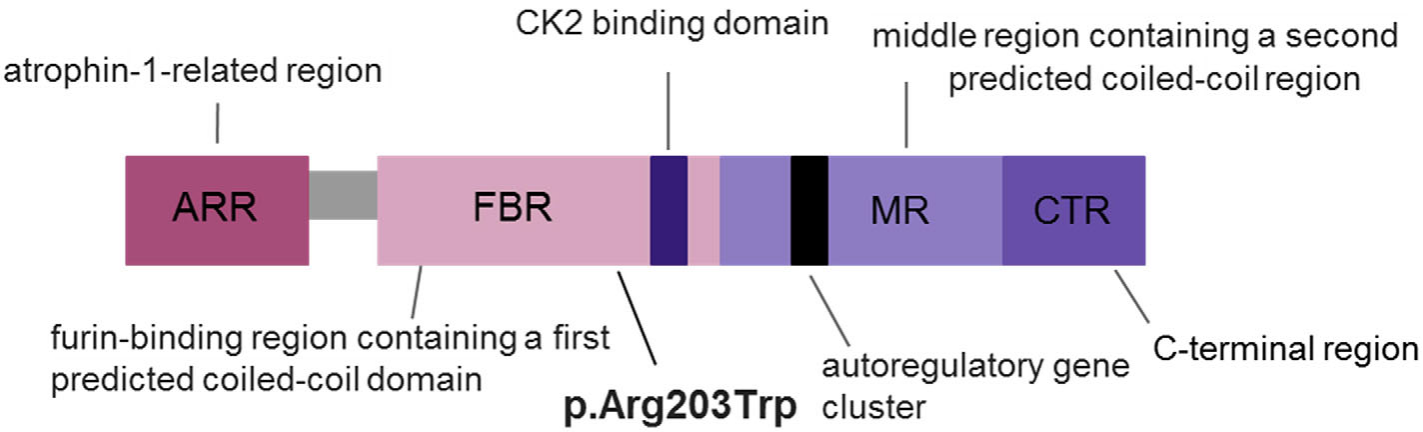
Phosphofurin Acidic Cluster Sorting Protein 1 (PACS1) organization

The typical phenotype of the SHMS is characterized by neurodevelopmental delay with variable severity, language delay, hypotonia, feeding difficulties, seizures, behavioral problems, congenital heart anomalies, short stature, and microcephaly (Tenorio‐Castaño et al., 2021). Language skills are more severely impaired compared to motor skills (Lusk et al., 2020). Hypotonia and feeding difficulty are common (Schuurs‐Hoeijmakers et al., 2016). Nevertheless, a series of unique clinical findings have been reported over the years such as retrognathia, absent nasal bone, and colpocephaly (Tenorio‐Castaño et al., 2021).

We present the first Italian patients with SHMS. Patient 1 was an 8‐year‐old boy born from healthy non‐consanguineous parents at the end of a pregnancy complicated by maternal hypertension that occurred in the last trimester. Gastroesophageal reflux and femoral fracture are reported during the perinatal period. At 7 months, head and trunk control had not yet been acquired. He started to walk with support at 3 years of age; his first words at 4 and a half years. He presented a hypertonic crisis in absence of fever, for which he began therapy with valproic acid. Parents reported a regression of his motor skills after the first critical episode. At the age of 7, he still did not speak and walk independently. He suffered from stypsis and followed a semi‐solid diet. He underwent bilateral orchiopexy for cryptorchidism. Electroencephalography (EEG) revealed a background slowing and evident multifocal paroxysmal activity. Cerebral magnetic resonance imaging (MRI) showed a mild deformation of the profile and flattening of the occipital horns of the lateral ventricles associated with slight altered signal intensity. His physical examination showed the following parameters: weight 23 kg (10th–25th percentile), height 138 cm (90th–97th percentile), head circumference (HC) 51 cm (10th–25th percentile). Peculiar facial characteristics were noted: low anterior hairline, synophrys, arched eyebrows, down slanted palpebral fissures, hypertelorism, bulbous nasal tip, flat philtrum, thin lips, spaced teeth (Figure 2).

**FIGURE 2:**
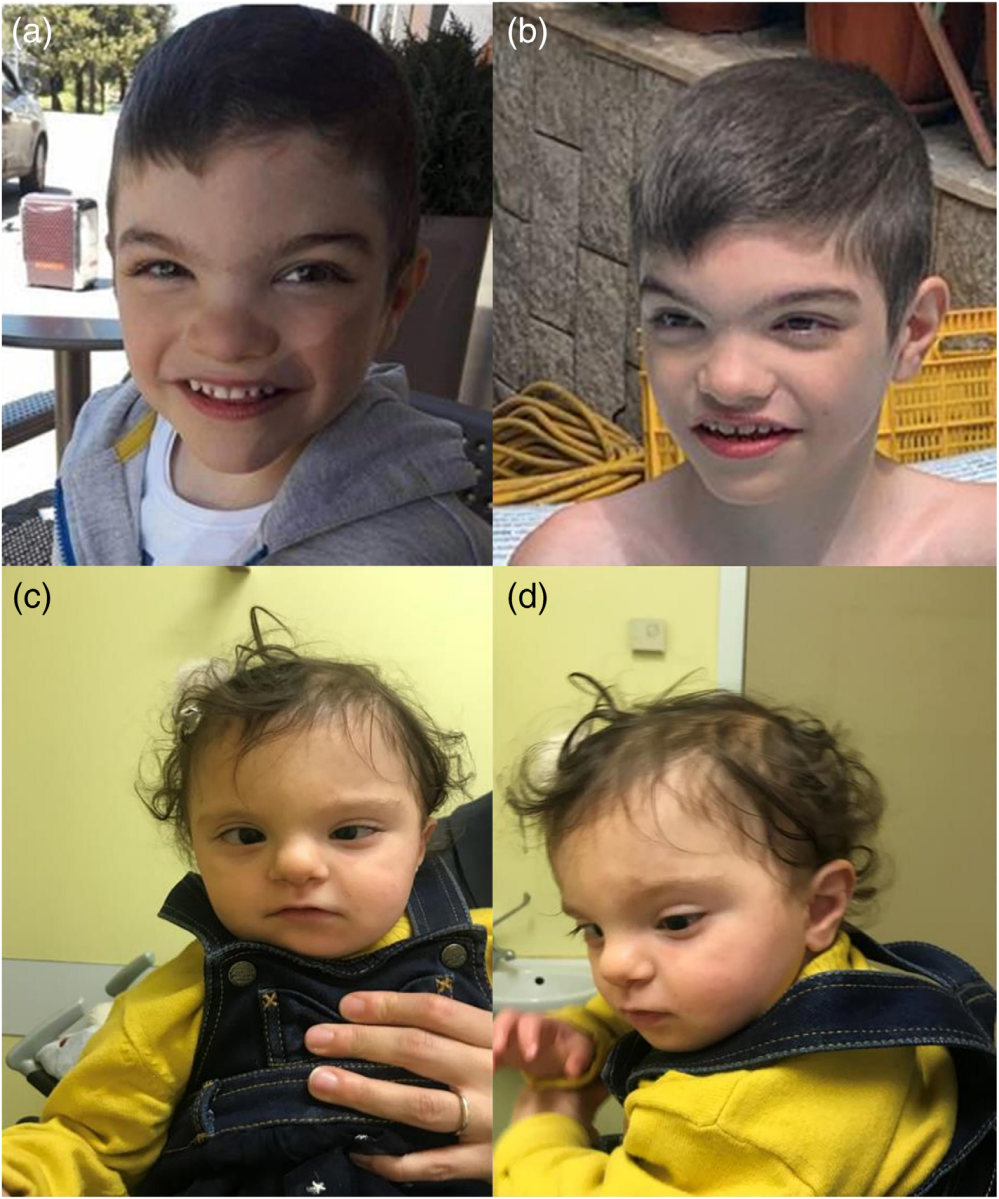
(a) Frontal view of patient 1 showing low anterior headline, synophrys, arched eyebrows, downslanting palpebral fissures, hyperthelorism, bulbuos nasal tip, flat philtrum, thin lips, and spaced teeth. (b) View of the three‐quarter profile of Patient 1. (c) Frontal view of Patient 2 showing the main clinical features: Thin and sparse hair, strabismus, epicanthus, high nasal bridge, prominent columella, and thin upper lip. (d) Lateral view of Patient 2 showing detached ear lobule

He showed bilateral clubfoot. He had hypertonic lower limbs and hypotrophy of muscle mass. He also presented bruxism, cold extremities, and hand‐stereotypies. His array‐CGH analysis did not reveal pathogenic alterations.

Patient 2 is a 1 year and 5‐month‐old girl, who was born at 36 weeks of gestational age by cesarean section due to placenta previa and labor onset. Her echocardiogram revealed a perimembranous ventricular septal defect with left to right shunt and muscular apical ventricular septal defect with left to right shunt. While EEG, abdominal echocardiography was normal. The ocular examination highlighted a bilateral retinal coloboma. Her neurological examination revealed hypertonia of the lower limbs, sitting position autonomously acquired, initial standing position with support. She showed interest in sound or color stimuli. Moreover, she manifested involuntary movements of the eyes: nystagmus, esotropia, saccadic movements, and she tended to keep her head forward. At the time of the evaluation, she started to walk with support and babble. She suffered from esophageal reflux and constipation.

Physical examination showed: weight of 7.3 kg (<3rd percentile), height of 72.5 cm (<3rd percentile), HC of 42.5 cm (<3rd percentile). She presented thin and sparse hair, strabismus, iris sizes' asymmetry, epicanthus, high nasal bridge, prominent columella, detached ear lobule, bifid tragus of the right ear, thin upper lip, downturned corners of the mouth, single palmar crease on the right hand, tapering fingers (Figure 2).

CGH‐array and analysis of *CDH7* gene did not reveal pathogenic alterations.

By exome sequencing (ES), we identified a missense variant c.607C>T p.(Arg203Trp) in exon 4 of the *PACS1* gene (NM_018026) in heterozygous state in both probands, which we next validated via Sanger (Figure 3). The effect on the encoded mutated protein has been predicted using CADD (combined depletion annotation depletion). The amino residue is evolutionarily conserved (Figure 3).

**FIGURE 3:**
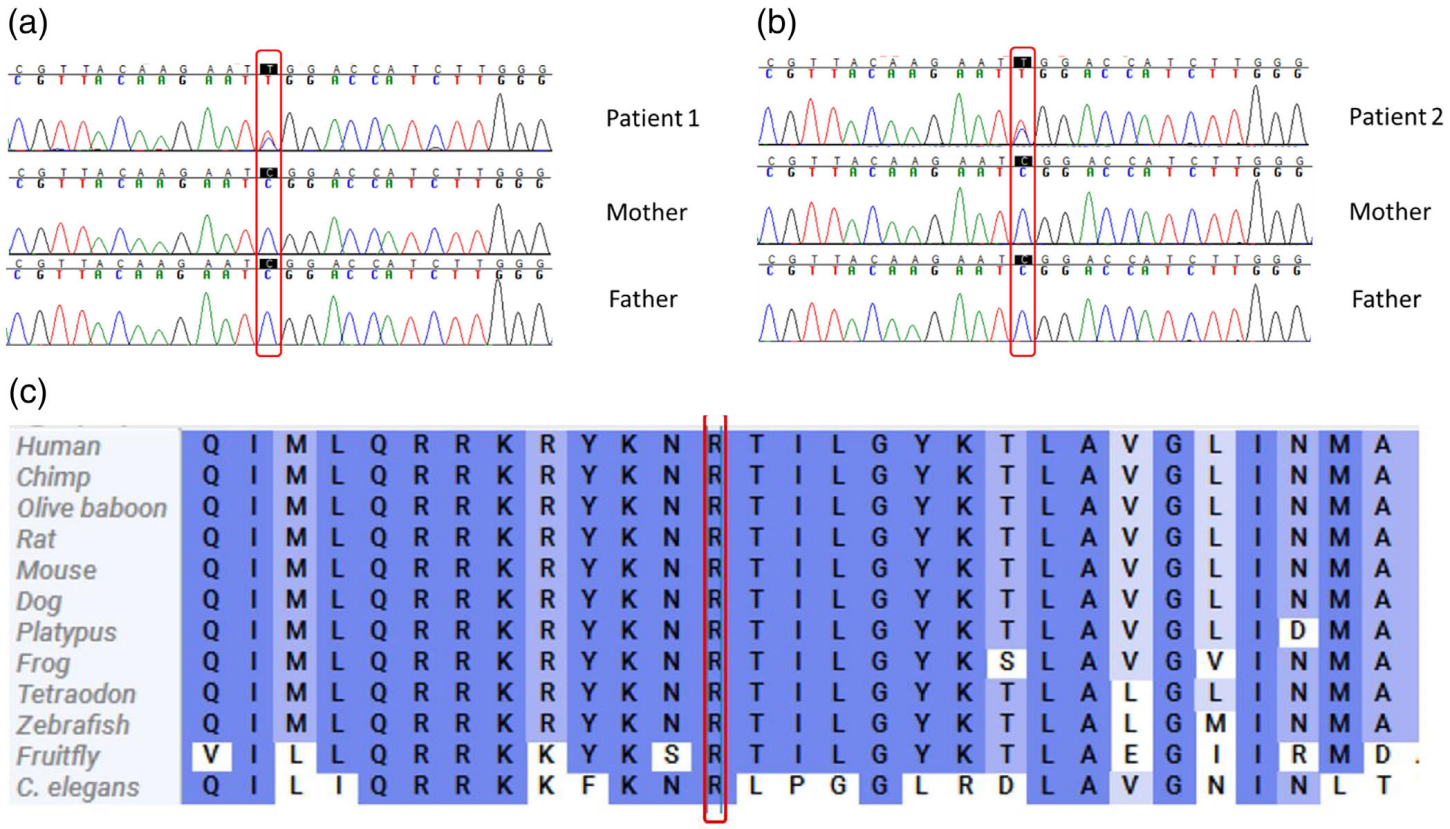
(a, b) Variant confirmation and segregation analysis by Sanger sequencing in Patients 1 and 2; **(c)** evolutionary conservation of the altered amino acid residue

Its reported MAF in gnomAD (https://gnomad.broadinstitute.org/) is 0.0008.

The variant c.607C>T p.(Arg203Trp) was recorded as rs398123009 in the dbSNP database (https://www.ncbi.nlm.nih.gov/snp/) with a pathogenic clinical significance due to in vitro and in vivo experiments demonstrating its damaging effects on the encoded protein. In particular, to understand the pathomechanism of the p.Arg203Trp hot spot variant, Schuurs–Hoejmakers and colleagues studied PACS1 in vitro and in vivo (Schuurs‐Hoeijmakers et al., 2012). Localization of GFP‐tagged wild‐type (p.Arg203) and altered (p.Trp203) PACS1 in transfected ARPE‐19 cells was evaluated to assess whether the phenotypes observed might be the result of misfolding and/or mistrafficking of the protein. As a result, altered PACS1 forms cytoplasmic aggregates in vitro with concomitant increased protein stability and shows impaired binding to an isoform‐specific variant of TRPV4. Furthermore, expression of mutant PACS1 mRNA in zebrafish embryos induces craniofacial defects most likely in a dominant‐negative fashion. This phenotype is driven by aberrant specification and migration of SOX10‐positive cranial neural‐crest cells suggesting that PACS1 is necessary for the formation of craniofacial structures. Furthermore, it has been reported in many individuals with intellectual disability with or without epilepsy, in a de novo status in the totality of the patients.

Patients with a different geographic origin manifest atypical trait that can mislead the medical diagnosis. In this perspective, molecular analysis employing exome sequencing can be supportive in the medical ascertainment of the SHMS in children. Individuals from four continents (Africa, Asia, Europe, America) have been collected. Among the published patients, whose ethnic origin is known, the presence of clubbed nails in a Dutch patient, pigmented nevi in a patient from Belgium, cleft lip in an Indian patient, involuntary movements and lipomyelomeningocele in two Japanese patients, hypogammaglobulinemia in a Turkish patient and absent gonadal development in a Chinese patient were reported as atypical clinical traits (Dutta, 2019; Hoshino et al., 2019; Liu et al., 2021; Lusk et al., 2020; Miyake et al., 2018; Schuurs‐Hoeijmakers et al., 2012; Schuurs‐Hoeijmakers et al., 2016; Scott et al., 2003; Simmen et al., 2005; van Nuland et al., 2021).

Our findings allow us to update the list of the SHMS clinical traits described so far. Intellectual disability (58/60), dysmorphic facial features (51/62), and speech delay (44/57) emerge as very frequent traits. Patient 1 suffered from seizures, a frequent clinical trait present in 34 out of 60 individuals, whereas motor delay (23/59), structural brain anomalies (15/51), and cryptorchidism (13/40) are reported as infrequent clinical features. Coloboma of choroid (6/51) consists of an SHMS rare clinical sign. Among the most unusual clinical characteristics (identified in 25% of the cases or less), down slanted palpebral fissures (15/62), bulbous nose (14/62), ocular hypertelorism (13/62), thin upper lip (13/63), gastroesophageal reflux (11/57), arched eyebrows (10/62), flat philtrum (7/62), low anterior hairline (4/62), synophrys (5/62), are reported (Tenorio‐Castaño et al., 2021). Interestingly, the boy presented bilateral clubfoot as a clinical feature first associated with SHMS, instead, the girl displayed coloboma of the choroid as a rare clinical feature.

In conclusion, this study allowed us to expand the phenotypic spectrum of the SHMS associated with the p.(Arg203Trp) mutation according to ethnic origin. Further studies are required to identify modifier genes that can modulate the clinical phenotype.

## AUTHOR CONTRIBUTIONS

Lucia Pia Bruno drafted the manuscript, made the experiments and contributed importantly to the interpretation of the results. Gabriella Doddato performed the experiments, made important contributions to the interpretation of the molecular results and drafted the manuscript. Margherita Baldassarri conducted the genetic counseling to the family and drafted the manuscript. Sara Resciniti performed the experiments. Mirella Bruttini and Lista Mirjam made contributions in the interpretation of the molecular results. Kristina Zguro and Simone Furini conducted the data acquisition. Maria Antonietta Mencarelli and Caterina Lo Rizzo conducted the genetic counseling to the family. Alessandra Renieri and Francesca Ariani have supervised and substantively reviewed the manuscript. All authors have read and agreed to the published version of the manuscript.

## CONFLICT OF INTEREST

The authors declare that they have no conflict of interest.

## ETHICS STATEMENT

The procedures employed agreed with the Helsinki Declaration's principles.

## INFORMED CONSENT STATEMENT

The parents of the patients provided their written informed consent to participate in this study.

## Data Availability

The data that support the findings of this study are available from the corresponding author upon reasonable request.
